# Nursing Care Across the Clinical Continuum of TAVI: A Systematic Review of Multidisciplinary Roles

**DOI:** 10.3390/jcm14134535

**Published:** 2025-06-26

**Authors:** Anna Jendrzejczak, Jadwiga Klukow, Joanna Czerwik-Marcinkowska, Wojciech Styk, Szymon Zmorzynski

**Affiliations:** 1 Chair of Nursing Management and Clinical Nursing, Institute of Clinical Sciences, Faculty of Health Sciences, Academy of Zamosc, 22-400 Zamosc, Poland; anna.jendrzejczak@akademiazamojska.edu.pl (A.J.); jadwiga.klukow@akademiazamojska.edu.pl (J.K.); 2 Institute of Biology, Jan Kochanowski University, 25-369 Kielce, Poland; marcinko@kielce.com.pl; 3 Academic Laboratory of Psychological Tests, Medical University, 20-059 Lublin, Poland; wojciech.styk@gmail.com; 4 Institute of Human Sciences, Faculty of Health Sciences, Academy of Zamosc, Pereca Street 2, 22-400 Zamosc, Poland

**Keywords:** aortic stenosis, cardiac surgery, nursing care, preoperative period, perioperative period, postoperative period, patient education

## Abstract

**Background/Objectives**: Aortic stenosis is a common heart disease, particularly among elderly patients. Transcatheter aortic valve implantation (TAVI) offers a minimally invasive alternative method to surgical valve replacement for high-risk patients. Although clinical guidelines for TAVI are well established, standardized nursing care pathways are lacking. This systematic review aims to clarify the nursing role in the pre-, peri-, and postoperative phases of TAVI. **Methods**: This review was conducted in accordance with the PRISMA guidelines. After applying the eligibility criteria, ten studies were selected from five databases: PubMed, Scopus, CINAHL, Web of Science, and the Cochrane Library. The work was registered in the PROSPERO database with the ID number CRD420251061863. **Results**: The analysis revealed the following: (1) a strong emphasis on preoperative patient education, often led by nurse coordinators; (2) perioperative nursing roles in conscious sedation protocols and early mobilization; (3) a lack of standardized rehabilitative protocols, especially in the preoperative phase; and (4) an emerging but insufficiently evaluated role of the TAVI nurse coordinator in multidisciplinary care. Most studies concentrated on postoperative care, outcomes, follow-up, and rehabilitation, but the small sample sizes limited the strength of the conclusions. **Conclusions**: Nurses play a vital role in multidisciplinary TAVI teams. There is an urgent need for evidence-based nursing guidelines to standardize care, improve clinical outcomes, and address the needs of TAVI patients. This review highlights the pivotal contribution of nursing to the success of TAVI.

## 1. Introduction

Cardiovascular diseases are the leading cause of death worldwide, and their incidence is increasing, especially in individuals over 60 years of age [1,2]. The most commonly diagnosed heart valve defects in Europe are aortic valve stenosis (aortic stenosis, AS) and mitral or aortic valve regurgitation [2]. It is estimated that in developed countries, 2–7% of the population over the age of 65 is diagnosed with valvular heart conditions [3,4]. The incidence of age-related AS has increased over the past two decades [5]. The extension of life expectancy has led to an increase in the number of patients with severe AS. These patients have an unfavorable prognosis if not treated surgically. Invasive treatment of AS improves the patients’ prognosis and quality of life. Owing to the very high risk during surgical procedures, one-third of patients with AS are disqualified from this type of treatment [6]. The perioperative mortality associated with isolated aortic valve replacement in patients younger than 70 years is 4–8%, and it increases to approximately 15% in patients older than 70 years [6]. An effective alternative treatment option for patients at very high surgical risk or patients not eligible for surgery is transcatheter aortic valve implantation (TAVI), known as transcatheter aortic valve replacement (TAVR) [6,7]. The minimally invasive nature of TAVI eliminates the need for sternotomy and cardiopulmonary bypass. It decreases the duration of surgery and anesthesia [8]. Elderly patients are eligible for TAVI surgery, so attention should be given to optimizing all stages of patient care, avoiding general anesthesia, minimizing procedural sedation, and standardizing perioperative management [9].

Guidelines for the TAVI procedure were established by the (I) European Society of Cardiology (ESC)/European Association for Cardio-Thoracic Surgery (EACTS) 2021 [10], American College of Cardiology/American Heart Association 2020 [11], and European Association of Percutaneous Cardiovascular Interventions [12]. These documents specify that decisions about the procedure and evaluation should consider valve viability, anatomic status, patient life expectancy, and patient preference. While the guidelines include a description of dedicated nursing staff with experience in caring for patients with valvular heart defects, they lack specific guidelines for nursing staff. The process of TAVI-related nursing practice involves the integration of medical, social, psychological, and other knowledge and skills in patient care. The friendly atmosphere in the ward, the open attitude of the staff, the use of therapeutic communication principles integrated into the patient care, and the individualization of activities are conducive to nursing’s therapeutic, counseling, and educational activities [13]. ESC and EACTS recommend that treatment decisions for patients with severe aortic stenosis be made by the Heart Team. This is a multidisciplinary team of specialists that comprehensively assesses patients, selects the best treatment option, and optimizes the entire process, from qualification to surgery to postoperative care. It includes interventional cardiologists, cardiac surgeons, clinical cardiologists, radiologists (or cardiologists), anesthesiologists, TAVI nurses/care coordinators, rehabilitation specialists, and geriatricians (optional) [10,11]. Nurses prepare patients for the TAVI procedure, including education, health assessment, and physical preparation (for example, shaving the insertion site), and provide psychological support [14]. Moreover, TAVI nurses assist in the hybrid (or treatment) room, monitor the patient’s condition in real time, and manage medications and equipment [15]. Proper qualification of the patient for TAVI and professional care by the interdisciplinary team at each stage of the procedure reduce the incidence of adverse symptoms [16]. The most common complications after TAVI include stroke, myocardial infarction, atrioventricular conduction disturbances, perivalvular leakage, local vascular complications, and embolization of the implanted valve prosthesis [17]. After the procedure, nurses are involved in the early detection of complications (e.g., bleeding, arrhythmia, infection), postinjection wound care (observation, dressing changes, assessment of bleeding or swelling), rehabilitation support, and information on follow-up care and education of the patient to pay attention to adverse symptoms after the procedure [18]. Owing to the complexity of the procedure, patients who undergo TAVI require specific pre-, peri-, and postoperative management. The present literature review was designed to collect and summarize information on nursing care in various periods of TAVI. Although numerous studies address the TAVI method, institutional care practices remain inconsistent [7,19,20]. In addition, most of the articles usually refer to nursing pre- or postoperative care for TAVI patients, and in these cases, a holistic approach to these patients is lacking.

## 2. Materials and Methods

The review was conducted in accordance with the Preferred Reporting Items for Systematic Reviews and Meta-Analyses (PRISMA) recommendations [21,22]. The work was registered in the PROSPERO database with the ID number: CRD420251061863.

### 2.1. The Search Strategy

Two databases were initially searched: PubMed and Scopus (between 1 January 2014 and 31 December 2024). In addition to the PubMed and Scopus databases, the CINAHL (Cumulative Index to Nursing and Allied Health Literature), Web of Science, and Cochrane Library databases were searched to ensure a broader capture of the relevant literature. The same search terms were adapted to fit the indexing systems of these databases. This expansion increased the pool of initial records by approximately 38%, enhancing the comprehensiveness of the review. The search strategy consisted of the following terms: (Aortic Stenosis) OR (Aortic Valve Stenoses) OR (Transcatheter Aortic Valve Replacement) OR (Heart Valve Prosthesis Implantation) OR (Stenos*s, Aortic Valve) OR (Valve Stenos*s, Aortic) OR (Stenos*s, Aortic) OR (Transcatheter Aortic Valve Implantation) OR (Implantation, Heart Valve Prosthesis) AND (Preoperative Care) OR (Care, Preoperative) OR (Care, Postoperative) OR (Care, Operative) OR (Preoperative Procedure) OR (Postoperative Procedure) OR (Operative Procedure) OR (Procedure, Preoperative) OR (Procedure, Operative) OR (Procedure, Postoperative) OR (Procedures, Preoperative) OR (Procedures, Postoperative) OR (Procedures, Operative) OR (Preoperative Procedures) OR (Postoperative Procedures) OR (Operative Procedures) OR (Preoperative Interview) OR (Preoperative Visit) OR (Postoperative Visit) OR (Preoperative Education) OR (Postoperative Education) OR (Patient Education) OR (Preoperative Teaching) OR (Postoperative Teaching) OR (Nurs* care) OR (Nursing). The search formula and wildcards were flexibly changed by combining subject terms and free words according to Tan et al., 2024 [7]. Following duplicate removal and relevance screening, the literature represented a diverse range of TAVI care practices in the preoperative, perioperative, and postoperative phases. A total of 10 studies were included after applying eligibility criteria across 5 databases: PubMed, Scopus, CINAHL, Web of Science, and the Cochrane Library. These included 3 systematic reviews and 7 original studies. Quality assessment, AMSTAR 2, and the Newcastle–Ottawa Scale revealed that 7 studies were of moderate to high quality, and 3 had minor methodological concerns (such as small sample sizes or limited nursing-specific endpoints).

### 2.2. Inclusion and Exclusion Criteria

The inclusion criteria were as follows: (I) adult patients undergoing TAVI; (II) publications available online or on platforms in pdf form; (III) nursing care of patients scheduled for TAVI; and (IV) studies related to the course of TAVI; (V) publications in English; (VI) papers published in the last 10 years.

The exclusion criteria included animal studies, preprints, articles published in a language other than English, articles with insufficient information or limited access to the full text, and works published before 2014. Scientific articles were preverified by reviewing titles, abstracts, and keywords.

### 2.3. Information on the Included Literature

**Figure 1:**
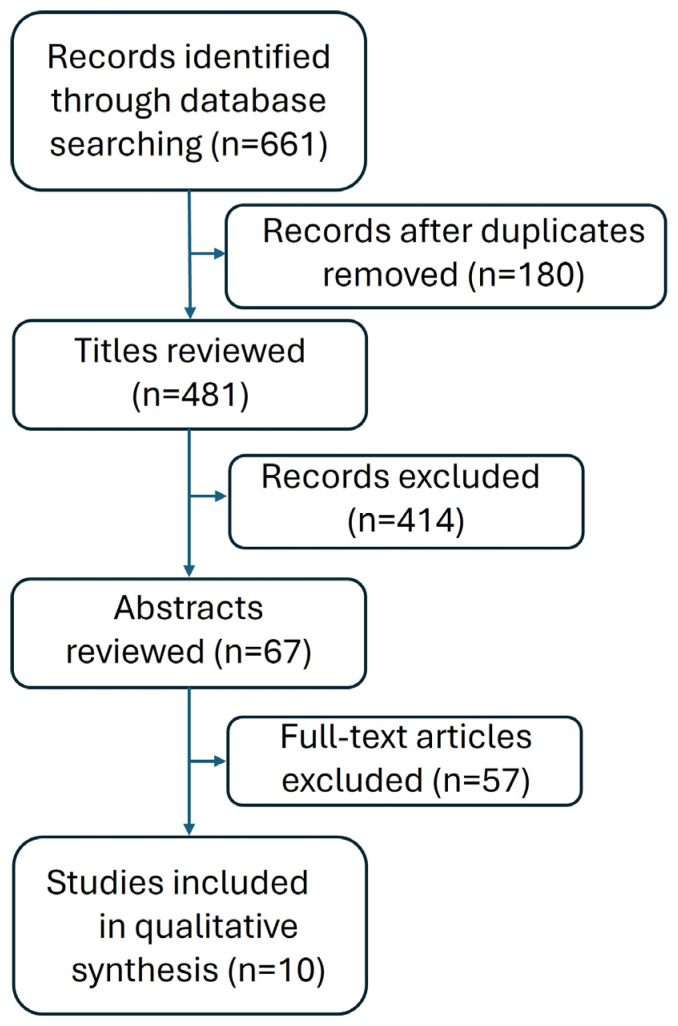
PRISMA flow chart for the literature search.

Titles and abstracts were assessed by two independent reviewers. Articles were excluded from the analysis if both reviewers agreed that they were ineligible. Both reviewers performed the final selection for full data extraction. This review yielded a total of 661 relevant literature items. After deduplication, 481 literature items were obtained. The PRISMA guidelines were used to select studies (Figure 1). After applying certain criteria, 10 articles were ultimately selected (Table 1).

### 2.4. Quality Assessment of the Included Studies

The methodological quality of the included studies was assessed via appropriate critical appraisal tools. For qualitative studies, the Critical Appraisal Skills Programme (CASP) checklist was applied. Each article was independently assessed by two reviewers, and discrepancies were resolved through discussion. Most studies were found to be of moderate to high quality, although a few had limitations related to sample size, reporting clarity, or lack of nursing-specific outcomes.

**Table 1:** Summary of studies associated with the care of TAVI patients.

No.	Author	Reference No.	Year of Publication	Number of Cases	Type of Publication	Analyzed Period	Main Findings	Nursing-Specific Findings
1.	Tan et al.	[7]	2024	Not applicable	Review	It refers mainly to the preoperative period.	The authors emphasize the crucial role of nursing care in the context of TAVI procedures, highlighting the need for evidence-based practice in the preoperative care of patients undergoing TAVI.	Nurses act as a vital liaison between the patient and the medical team, ensuring that information and recommendations are tailored to the individual clinical and psychosocial needs of the patient. As integral members of the Heart Team, nurses conduct comprehensive preoperative assessments including physical, psychological, and social evaluations. They provide patient-centered education, reinforce adherence to perioperative care plans, and identify potential barriers to optimal recovery. Nurses monitor patient understanding, emotional status, and readiness for surgery and contribute nursing-specific findings to interdisciplinary discussions to guide personalized care planning.
2.	Zou et al.	[23]	2023	Not applicable	Review	It refers mainly to the preoperative period.	The study provides valuable insights into the impact of cardiac rehabilitation on outcomes for patients undergoing TAVI, offering nurses evidence to enhance quality of care and improve patient results.	Nurses are integral members of the Heart Team. They perform comprehensive preoperative patient assessments that include patient education tailored to individual needs. Nurses take detailed medical, functional, and psychological history, identify nursing-specific risk factors, and use standardized risk-assessment tools. They evaluate the patients’ functional capacity, cognitive status, emotional well-being, and readiness for surgery. Nursing findings contribute directly to the interdisciplinary plan of care, highlighting patient strengths, needs, and potential barriers to recovery.
3.	McCalmont et al.	[24]	2021	2400	Original research	It refers mainly to the preoperative period.	The article describes the organization of TAVI patient care within the BENCHMARK international registry, highlighting efforts to standardize care quality and resource efficiency. It discusses the multidisciplinary care structure and processes, recognizing the important role of nurses, even though they are not the primary focus.	Recommendations for nurses on preoperative care for TAVI—assessing, educating, and preparing the patient for the procedure. The results of these nursing assessments and observations are an integral part of the Heart Team’s plan of care, enabling personalized patient preparation for the procedure.
4.	Kočka et al.	[25]	2022	128	Original research	It refers mainly to the perioperative period.	This article describes experiences with a nurse-led sedation (NLS) protocol during TAVI, demonstrating the feasibility and potential benefits of nurse-driven sedation management.	Nurses can safely and effectively provide sedation during TAVI and optimize early care after the procedure.
5.	Tanner et al.	[26]	2024	Group A—59 Group B—268 Group C—736	Original research	It refers mainly to the perioperative period and partially to the postoperative period.	The article highlights the important role of nurses responsible for data entry into the Mater TAVI database, emphasizing their contribution to data management and quality assurance in patient care.	These findings are important for nurses, helping them better monitor patients after the procedure and understand potential complications. The article can also enrich nursing education on advanced cardiac procedures.
6.	Panos and George	[27]	2014	Not applicable	Review	It refers mainly to the postoperative period.	The work discusses in detail the nurse’s vital role in monitoring and managing patients after TAVI, emphasizing its importance within the multidisciplinary patient care team.	Nurses caring for TAVI patients must understand the potential complications, and the impact of comorbid conditions on recovery. Specialized education is essential, as TAVI requires different nursing skills compared with traditional cardiothoracic surgery.
7.	Lysell and Wolf	[28]	2021	14	Original research	It refers mainly to the postoperative period.	The study highlights patients’ experiences of daily life before and after TAVI, emphasizing the importance of these insights for nurses in planning and personalizing patient care.	It relates to patients’ experiences, which are relevant to nursing in the context of mental support and rehabilitation. The nurse plays an important role in supporting the patient in regaining his or her independence and in preventing feelings of loneliness that may arise after surgery. Interventions should reinforce the patient’s sense of coherence and ability to rebuild strength, which helps him or her find his or her way in a new life situation.
8.	Lauck et al.	[29]	2020	Not applicable	Original research—recommendations for programs	It refers mainly to the postoperative period.	The study highlights the pivotal role of the nurse leading the TAVI program as a central coordinator and communicator.	The TAVI program nurse provides seamless communication with the multidisciplinary team, is an essential central point of coordination to manage individual patient cases, and is essential to inpatient assessment, education, discharge planning, and monitoring.
9.	Baumbusch et al.	[30]	2018	31	Original research	It refers mainly to the postoperative period.	The research provides valuable insights into patients’ experiences after TAVI and underscores the importance of the nurse’s role in delivering comprehensive care during the recovery period.	Nursing interventions should extend beyond the early weeks after surgery with consideration of patients’ age and disease context and caregiver involvement in the recovery process.
10.	Egerod et al.	[31]	2015	54	Original research	It refers to the postoperative period.	The article details patients’ immediate post-TAVI reactions, highlighting the important aspects of nurses who monitor the patient’s condition.	The nurse’s responsibilities include monitoring pain levels, providing an environment conducive to rest, nutritional protection (for nausea and vomiting), monitoring vital signs, reporting local bleeding, supporting the patient in early mobilization, educating the patient and family, as well as working as a part of the Heart Team.

## 3. Results and Data Presentation

The analysis highlighted the following: (1) a strong emphasis on preoperative patient education, often led by nurse coordinators; (2) perioperative nursing roles in conscious sedation protocols and early mobilization; (3) the lack of standardized rehabilitative protocols, especially in the preoperative phase; and (4) a growing but underevaluated role of the TAVI nurse coordinator in multidisciplinary care pathways. This review underscores the critical importance of structured nursing care in all stages of TAVI. Three articles focused on the preoperative TAVI period (Table 1). The goal of preparing patients for surgery is to perform the surgical procedure safely and reduce the risk of complications. While the intervention itself has become highly standardized, the nursing care practices surrounding it remain inconsistent. The expanded search revealed a modest yet consistent body of evidence supporting nurse-led initiatives across the care continuum. Two articles referred to the perioperative period, one of which considered this period exclusively (Table 1). Articles published 10 years ago pointed to the advantages of TAVI over surgery, which now seems obvious [32,33]. The articles related to the perioperative period focused on programs to increase the quality of care [24], treatment, and sedation [25].

Despite advancements in procedural outcomes, there remains a notable lack of research into standardized prehabilitation programs, the clinical effectiveness of TAVI nurse coordinators, and the long-term impact of structured discharge planning led by nursing staff. Establishing uniform, evidence-based nursing guidelines and enhancing interdisciplinary integration, particularly involving nurses in preoperative and rehabilitative roles, could substantially improve patient outcomes and procedural safety. Some articles were excluded from the analysis. For example, Picou et al. did not focus specifically on patients undergoing TAVI, which may limit its direct applicability to this context [34]. Although the article does not focus directly on the role of the nurse, its findings may inform patient needs that nurses can address in clinical practice, especially in the context of TAVI. Another example is the work of De Ronde-Tillmans et al. [35]. The article presents a comprehensive TAVI Care and Cure program that covers all stages of patient care, from qualification to hospitalization and postoperative follow-up. The program is designed to improve the quality of care and outcomes for patients undergoing TAVI. It involves the collaboration of various specialists, including cardiologists, cardiac surgeons, anesthesiologists, and geriatricians, which emphasizes the importance of a multidisciplinary team in TAVI patient care [35]. However, the article does not focus directly on the nurses’ role in TAVI. A patient undergoing TAVI is provided with professional interdisciplinary care from the time of admission to the department, through referral to the operating theatre, through performance of the operation, until the patient is transferred to the postoperative cardiac surgery unit, and then, until the day of discharge from the hospital [35]. Most of the articles focused on the postoperative period and dealt with outcomes, follow-up, and rehabilitation (Table 1). However, in most studies, the number of individuals in the study groups was relatively small, which may be a significant limitation in drawing appropriate conclusions.

### 3.1. TAVI Programs

TAVI places a heavy burden on patients, not only physically, but also mentally, and it triggers a high level of anxiety [36]. In addition, programs are being developed to appropriately address the patients from time of diagnosis through treatment and discharge from the hospital. One such program was the BENCHMARK [24]. It was created as a noninterventional, multicenter international registry. A total of 2400 patients with severe AS in 30 European centers were included. The implementation of these practices in 28 centers in Europe has led to a reduction in the length of hospitalization and intensive care unit stay while maintaining patient safety [37]. To achieve standardization of the quality of care, some centers have introduced dedicated TAVI coordinators or nurses to ensure a smooth, streamlined care pathway for all patients. The nurse coordinator plays a key role in ensuring continuity of care, especially for patients with chronic cardiovascular conditions. Tasks performed by the coordinator include monitoring patients’ health status, education, coordination of the medical team, and emotional support for patients and their families [24]. Through these activities, it is possible to improve treatment outcomes, reduce hospitalizations, and improve patients’ quality of life. In this context, the role of such a coordinator is becoming increasingly important, especially as the number of patients with chronic diseases increases [24]. In addition to the BENCHMARK registry, other programs such as the Edwards Benchmark Program, developed by Edwards Lifesciences, exist. This is an evidence-based program designed to optimize all stages of the TAVI clinical pathway: before, during, and after the procedure [38]. The program also supports training for nurses and TAVI coordinators and promotes the exchange of best practices between centers. Although the idea of the BENCHMARK registry was a very good concept, McCalmont et al. noted the lack of standardized procedures, assessment tools, educational materials, and available 20G needles; the high workload of nurses; and the lack of time for detailed patient care [24]. In addition, nurses are not always aware of the actual benefits of each intervention. Another aspect is the differences in patients’ willingness to be educated, due to age or education, which requires family involvement in the educational process during preoperative visit-care [24].

### 3.2. Preoperative Visit-Care

Tan et al. focused on the evidence for preoperative visit-care for TAVI and evidence-based support for clinical intervention [7]. In the study by Lyssel and Wolf, patients who understood their poor prognosis had no doubts about their decision to undergo TAVI later [28]. During the preoperative visit, it is necessary to prepare an appropriate surgical plan suitable for the patient [7]. Nurse-led programs can provide significant clinical benefits, such as improved heart rhythm control, reduced hospitalizations, and improved quality of life for patients [7]. Nurses play a key role in managing the care of patients, offering education, health monitoring, and care coordination [7]. Similar results were reported by Tong et al. [39]. With the advent of TAVI, a nursing care pathway including preprocedure care, care in the cardiac catheterization laboratory, postprocedure care, and cardiac rehabilitation began to be developed. Gibbins et al. emphasized proper patient education, such as through the development of a patient information booklet, to provide patients with the necessary information on a consistent basis [14]. An example of an activity that requires appropriate recommendations for nurses is preoperative TAVI rehabilitation [23]. Zou et al. emphasize the importance of an interdisciplinary approach in cardiac rehabilitation, which often involves the collaboration of specialists from different fields, including nurses [23]. On the basis of their review paper, conclusions can be drawn about the possible participation of nurses in cardiac rehabilitation, including (I) patient education by providing information on the TAVI procedure, preparation for the procedure, and postoperative management [40]; (II) health monitoring including assessment of vital signs, identification of risk factors, and adjustment the plan of care according to the patient’s condition [41]; and (III) psychological support by helping the patient cope with the anxiety and stress of the upcoming procedure [42]. Authors Tan et al. and Zou et al. highlighted the role of nurses in the multidisciplinary team performing the TAVI procedure as well as the importance of nurses in educating patients before the procedure and preparing patients for the procedure [7,23]. Much has been written about the importance of nurses in the TAVI preoperative period, but this must be followed by systemic changes. Tan and colleagues [7], similarly to McCalmont et al. [24], reported a lack of standardized procedures, insufficient educational materials, a shortage of training, workload, and limited psychological support [7]. Conducting a structured preoperative visit allows for an accurate assessment of patients’ physical and mental health before surgery [43].

### 3.3. Nurse-Led Sedation

Nurses’ participation in vascular ultrasound management and vascular access selection is an important step in optimizing care for patients undergoing cardiac procedures, including TAVI. Studies indicate that nurses who specialize in ultrasound techniques can perform vascular assessments effectively, which contributes to the selection of the safest and most appropriate access route, reducing the risk of vascular complications and improving procedure outcomes [44]. In addition, in many centers, nurses are actively involved in the valve installation procedure, providing precise equipment preparation and operator support [45]. This physician–nurse collaboration and increased nurse competence contribute to streamlined procedures and increased patient safety [46]. The implementation of standards and/or protocols for nurses’ participation in vascular ultrasound and “valve handling” is therefore recommended as part of modern cardiac care.

The number of TAVI procedures performed under local anesthesia with or without conscious sedation is increasing [47]. Patient predispositions and other factors, as well as the experience of the center and the operator, should play a major role in deciding on the optimal type of anesthesia [48]. Local anesthesia–sedation can be safely performed during TAVI and may be the first option for suitable patients because of the shorter surgery duration [49]. A study by Cereda et al. revealed that the nurse, through access to the distal radial artery, could independently perform key procedures such as puncture and wire and catheter insertion under the supervision of an interventional cardiologist [50]. Despite the technical difficulty of this method, the nurse achieved a high success rate (89%) with a low complication rate, and improvement in outcomes was evident with experience (learning curve) [50]. The nurse’s involvement in this type of procedure is promising, benefiting both staff and patients, and points to the possibility of expanding nurses’ tasks in the catheterization room. Instrument nurse care in the operating room increases patient confidence and reduces anxiety levels [7]. In the study by Kocka and colleagues, the anesthesia team consisted of a certified anesthesiologist and a nurse [25]. A dedicated team of catheterization laboratory nurses was trained in sedation by an expert anesthesiologist. The nurse administering sedation during TAVI was fully focused on assessing the patient and had no other responsibilities during the procedure [25]. However, this study was nonrandomized and conducted at a single, experienced center. In addition, the selection of patients for the nurse-led sedation group did not meet strict criteria. Kocka and colleagues concluded that important factors in the choice of sedation were associated with procedural difficulties due to peripheral vascular disease or large prosthesis size [25]. Furthermore, they reported that clinical outcomes and complications were similar with or without the presence of an anesthesiologist during the procedure, and nurse-led sedation may result in shorter intensive care stays and cost savings [25]. The development of specific guidelines and multidisciplinary risks can improve TAVI patient care [51]. The surgery time and hospital stay were longer in patients who underwent the procedure under general anesthesia than in those who were sedated [33,52]. The role of the nurse is to carry out the procedures for preoperative and postoperative management, providing the patient with relevant information in this regard [35,49]. Among other things, the anesthesia nurse should conduct direct observation of the patient, correctly interpret test results, and control vital parameters.

Articles on the perioperative period mostly address the course of the surgery itself, the prostheses used, and the anesthetics used and do not focus directly on the tasks of nurses [32,33,49,53,54]. Nurses are responsible for patient safety during surgery and must be ready for a dynamic influx of patients and the provision of quality care with higher workloads [26]. Although the study by Tanner and colleagues is not randomized, they provide specific recommendations for nurses involved in TAVI [26]. These factors are related to patient management (mainly related to psychological support), sedation training, standardization of patient management at discharge, and information transfer in the Heart Team [26].

### 3.4. Outcome

An exponential increase in the number of TAVI procedures has been observed along with a growing role for nurses in the process [26]. After the TAVI procedure, the patient should be cared for in a postoperative or cardiac intensive care unit [55]. Owing to the risk of arrhythmia, the patient is temporarily implanted (for 48 h) with a right ventricular pacing electrode [56]. The first 24 h after surgery are crucial, and patients’ condition should be monitored, with particular attention given to water balance, electrocardiogram results, hemoglobin levels, and renal function [56]. The development of care protocols is valuable for guiding nurses in time for emergency events. Early recognition of dysrhythmias with the effective use of transvenous stimulation, hemodynamic management, detection of subtle neurological changes, and assessment of hematomas at the surgical site are key areas in which nurses provide first-line care and intervention [27]. Suboptimal TAVI valve performance (i.e., prosthesis mismatch and bicuspid regurgitation) was associated with an increased risk of adverse clinical events within 30 days of noncardiac surgery [54]. A study by Khan et al. revealed that the presence of cognitive deficits predicted the occurrence of postoperative cognitive dysfunction and mortality after TAVI, highlighting the value of screening for geriatric risk factors before surgery to identify high-risk patients [57]. A retrospective study by Ebrahimian and coauthors evaluated the risk of postoperative complications after noncardiac surgery (NCS) performed in patients who had previously undergone TAVI [51]. Their results suggest that the interval between TAVI and NCS is not a predictor of complications [51]. Makkar and co-authors showed no significant difference in the incidence of death or disabling stroke 5 years after TAVI compared with surgical aortic valve replacement in patients with AS who were at moderate surgical risk [58].

Patients’ daily lives before and after TAVI were analyzed, including assessments of changes in function, quality of life, and perceptions of the procedure [28]. The article by Lyssel and Wolf [28] does not directly address nurse-led sedation, nursing tasks during surgery, or formal nursing protocols for perioperative sedation. However, it highlights areas where nursing care can be enhanced, including providing emotional support before surgery to reduce anxiety, offering postoperative education on recovery and warning signs, developing individualized patient care plans, and ensuring ongoing monitoring of the patient’s condition after discharge [28]. Attention is being given to the role of cardiac nurses in the postoperative period [29]. Egerod and colleagues emphasized the need for an evidence-based management pathway and highlighted the importance of nonpharmacological and pharmacological nursing interventions [31]. These findings demonstrate the practical application of the nursing role in comprehensive care after TAVI [31]. Baumbusch et al. focused on patients’ experiences one year after TAVI, emphasizing the importance of continuity and comprehensiveness of care [30]. They noted the need to consider the patient’s perspective when planning care and the nurse’s role in providing emotional support, education, and health monitoring in the long term after surgery [30]. However, this study has several limitations. It was conducted at a single TAVI center, and the sample size was small, which limits the overall ability to generalize the effects to broader populations. In addition, the interviews took place one year after the procedure. The participants may not have remembered all the details or may have interpreted events from the perspective of past time.

### 3.5. Nurse-Led Mobilization

The goal of rehabilitation is to achieve independence and self-sufficiency and counteract the effects of immobilization as soon as possible. A standard protocol of 4 h of bed rest followed by nurse-led mobilization at least twice on the day of surgery and a demonstrated return to baseline mobilization the morning after surgery facilitates the goal of being ready for safe discharge the next day [59]. Nurse-led mobilization 4 h after TAVI is feasible for most patients who receive follow-up care and is an important intervention to reduce the risks associated with treating patients with valvular heart disease [59]. Patients should be encouraged to mobilize early unless there is hemodynamic instability; vascular complications; little or no urine output; unresponsiveness to diuretic therapy; or significant changes in hemoglobin levels, renal function, and neurological status. Regularly scheduled exercise, along with other interventions such as pharmacology, is a recognized component of cardiac rehabilitation and can significantly complement the effects of TAVI [23]. Several studies have shown that intensive postoperative recovery programs, such as short-term exercise-based cardiac rehabilitation, especially in Phase I during hospitalization, can increase functional capacity, quality of life, and exercise tolerance [60,61,62]. However, heterogeneity in rehabilitation protocols has been observed. The speed of improvement depends on the patient’s general condition and the occurrence of any complications [63]. Exercise should be discontinued when symptoms arise such as coronary pain, shortness of breath, acceleration of heart rate by more than 20 beats/min, slowing of heart rate by more than 10 beats/min, exercise-induced arrhythmia, blood pressure values decreased by more than 10–15 mmHg, systolic pressure increased by more than 40 mmHg, and/or diastolic pressure increased by more than 20 mmHg from baseline. It is recommended that the exercises be repeated twice a day. To maintain the continuity of rehabilitation, exercise should be performed on all days of the week [63]. A patient’s physical condition is significantly related to the prognosis after TAVI [23].

## 4. Discussion

In recent years, the number of TAVI procedures has increased exponentially. Key changes in TAVI practices have included an increase in the percentage of patients treated under conscious control and using transfemoral access. Significant reductions in procedure duration and length of hospital stay have been observed over time. This paper presents the importance of nurses during TAVI at different stages of this procedure. Most studies emphasize the importance of patient education (before the procedure) [7,23,24] as well as the role of nurses in the Heart Team [7,24,29]. However, it seems that TAVI nurse coordinators are underutilized. Few papers focus on the importance of nurses during this procedure, as reflected by the number of publications included in this review.

One of the risk factors associated with TAVI is patient age. Therefore, the emotional needs of elderly patients should also be considered. Maintaining patient autonomy is one of the key ethical challenges in caring for older adults with multimorbidity [64]. A study by Alodhialah and coauthors highlighted the important role of nurses in managing the ethical and legal challenges of caring for older adults with multimorbidity [65]. Meeting these challenges requires ongoing education, appropriate communication, and policies designed to empower nurses. Nurses point to difficulties in obtaining informed consent from older people with cognitive impairment. This is a major limitation in understanding the provided information and raises a dilemma between patient autonomy and patient safety. Autonomy is a fundamental ethical principle in health care, affirming patients’ right to make informed decisions about their care [66]. They note the need to adjust communication (language, form) and often include caregivers or proxies with patients with impaired decision-making capacity [65]. Nurses report on the complex ethical and legal challenges of accessing and complying with the wishes of patients in the terminal phase, including drafting decisions to opt out of interventions [65]. They play a crucial role in supporting and advocating for the needs and preferences of older adults living with multiple morbidities [67]. Meeting the above functions requires a well-crafted strategy that combines ethical guidelines with legal obligations and professional standards.

### 4.1. Implications for Clinical Practice

Considering the potentially serious consequences associated with advanced AS, patients undergoing TAVI require active and multidisciplinary support from medical personnel, including nurses. Early identification of peri- and postoperative complications is crucial, as prompt intervention can prevent further health problems. High-risk patients should receive regular monitoring of vital signs, assessment of physical capacity, and education on lifestyle and medication intake. In addition, the health care system should focus on patient- and family-centered care considering psychosocial challenges.

### 4.2. Gap in the Literature

While the role of TAVI coordinators is emerging, empirical studies evaluating their clinical impact are scarce. Although De Silva and colleagues developed a post-TAVI nursing care protocol, other documents are lacking in this area [68]. Other gaps in the literature include a lack of preoperative education provided by nurses and heterogeneous nursing supervision coordination after TAVI. Variety is also observed in rehab protocols. It would be interesting to investigate whether preoperative cardiac rehabilitation can affect TAVI prognosis, which could increase the number of patient referrals for such rehabilitation procedures. Currently, there is insufficient evidence on the effectiveness of such preoperative rehabilitation [23,69]. However, Weber et al. reported that preoperative inspiratory muscle training (IMT) significantly improved inspiratory muscle function and reduced the number of patients who developed pneumonia by 75% and the length of hospitalization among patients who underwent perioperative IMT by 25% [70]. IMT is an important activity performed before surgery that can reduce the incidence of postoperative pulmonary complications. In addition, among younger, low- to intermediate-risk TAVI patients, IMT has also been shown to facilitate recovery from surgery and to improve cardiac minute volume and inspiratory muscle strength [71,72]. IMT improved exercise tolerance, lung ventilation function, and inspiratory muscle strength; shortened the postoperative hospital stay; and reduced postoperative complications in patients after TAVI. All these results appear to have a lasting effect on improving survival time [73].

Furthermore, the psychological needs of elderly patients after TAVI (e.g., depression, isolation) are well documented, but the role of nurses in addressing these needs has not been well studied.

### 4.3. Limitations of the Included Studies

The included studies present several limitations. Many of these studies were based on small patient cohorts and were conducted as single-center investigations where TAVI procedures were performed [25,28]. Moreover, the studies lacked randomization, as patient allocation was determined by the order of enrollment rather than through random assignment. All patients received treatment at high-volume centers with extensive experience in TAVI procedures, which may limit the generalizability of the findings to settings with lower procedural volumes or less-experienced operators. Most interviews were conducted within six months postprocedure; thus, a longer follow-up period might have captured changes in patients’ experiences and challenges, including those related to rehabilitation and mental health.

### 4.4. Limitations of the Review Process

The review process was limited by the strict inclusion and exclusion criteria, such as the restriction to English-language publications and studies published within the last 10 years. This may have led to the omission of relevant data from older studies or research published in other languages. Furthermore, preselection on the basis of only titles, abstracts, and keywords might have resulted in the exclusion of valuable studies that did not clearly reflect their relevance in these sections.

### 4.5. Recommendations

Considering the selected articles, we made recommendations for the nursing care of TAVI patients and their implementation strategies. We prepared a step-by-step action plan that included the preprocedure and immediate perioperative period (short-term actions), the postoperative phase and discharge (medium-term actions), and postdischarge follow-up and extended recovery (long-term actions).

Short-term actions performed by TAVI nurses include (I) conducting a comprehensive qualifying assessment that considers the patient’s clinical, functional, and psychosocial status as well as individual needs, taking into account age and comorbidities; (II) providing appropriate preoperative education to the patient and family regarding the procedure, possible complications, course of hospitalization, and expectations for recovery; (III) offering emotional and psychological support prior to the procedure to help prepare the patient mentally for TAVI; (IV) planning preprocedure logistics to minimize hospital visits and optimize the diagnostic pathway; (V) ensuring effective communication within the multidisciplinary team (Heart Team) to enable timely decision making during the procedure; and (VI) providing intraoperative support and continuous monitoring of the patient’s condition during TAVI.

The medium-term actions may include the following: monitoring the patient’s clinical condition and detecting complications after procedure, initiating rehabilitation as soon as possible after surgery, providing education on follow-up care, and developing an individualized discharge plan (which includes home care instructions and coordination with rehabilitation services).

Actions such as ongoing educational and psychosocial support, including involving the patient in decision making related to recovery, monitoring clinical outcomes and functional status, and assessing self-care abilities and rehabilitation progress, are provided long-term.

These recommendations can be the basis for building high-quality nursing care at each stage of TAVI treatment, emphasizing the key role of nurses in the interdisciplinary team.

### 4.6. Conclusions

The following conclusions were drawn from our review: (I) standardized guidelines for comprehensive nursing care around TAVI are insufficient. Effective care for TAVI patients requires integrated, standardized programs and a coordinator (nurse) who provides continuity of care, education, emotional support, and coordination of the Heart Team. While initiatives such as the BENCHMARK and Edwards Benchmark Program provide benefits (including reduced hospitalizations and improved quality of care), significant challenges remain, such as the lack of standardized procedures, educational materials, and time and staffing constraints (nursing workload). (II) Effective preoperative care requires nurses’ active participation in education, health monitoring, psychological support, and coordination of care within the Heart Team. Organizational changes and the development of training programs are needed to fully realize the potential of nurses in improving the quality of care for patients undergoing TAVI. (III) TAVI procedures performed under local anesthesia with conscious sedation are increasingly safe and lead to shorter procedure times and hospitalizations. Nurses play a key role in perioperative care, providing real-time monitoring of the patient’s condition and psychological support. Specialized training and the development of standards and/or guidelines regarding the importance of nurses in sedation and care during TAVI can improve the quality of care and patient safety. The above measures are essential for optimizing the procedure and patient outcomes. (IV) Too little is still known about the effectiveness of preoperative rehabilitation. (V) After TAVI surgery, nurses play a key role in monitoring the patient’s condition, identifying complications early, providing emotional support, and educating patients in the postoperative period and during recovery. It is important to consider the individual needs of patients. (VI) There is a need for a holistic approach to TAVI patients that is not focused only on pre- or postoperative care.

## Data Availability

The original contributions presented in the study are included in the article, further inquiries can be directed to the corresponding authors.

## Abbreviations

The following abbreviations are used in this manuscript:

AS	Aortic stenosis
ESC	European Society of Cardiology
EACTS	European Association for Cardio-Thoracic Surgery
SAVR	Surgical Aortic Valve Replacement
TAVI	Transcatheter Aortic Valve Implantation
TAVR	Transcatheter Aortic Valve Replacement
